# Challenging the Comparison in Montgomery Between Patients and ‘Consumers Exercising Choices’

**DOI:** 10.1093/medlaw/fwab031

**Published:** 2021-09-09

**Authors:** Emily Jackson

**Affiliations:** Law Department, London School of Economics and Political Science, Houghton Street, London WC2A 2AE, UK

**Keywords:** consumers, disclosure, duty of care, informed consent, *Montgomery*, negligence

## Abstract

In *Montgomery v Lanarkshire Health Board*, Lords Kerr and Reed referred to the increasing tendency to treat patients ‘as consumers exercising choices’. The question of whether it is helpful to regard patients as consumers is not a new one, but it arises most frequently in discussions about the commercialisation of healthcare. Comparing patients with consumers in relation to informed consent is an interesting development, especially in the light of the growing body of contract and consumer law scholarship which questions the extent to which information disclosures to consumers produce informed choices. If there is evidence that the duties of disclosure which are imposed on retailers, in order to redress the imbalance of knowledge and power in the consumer–retailer relationship, do not always fulfil their intended purpose, might this have any resonance for the duties of disclosure which are imposed upon healthcare professionals?

## I. INTRODUCTION

In *Montgomery v Lanarkshire Health Board* (*Montgomery*),^1^ Lords Kerr and Reed referred to the increasing tendency to treat patients ‘as consumers exercising choices’. The question of whether it is helpful to regard patients as consumers is not a new one, but it arises most frequently in discussions about the commercialisation of healthcare.^2^ Comparing patients with consumers in relation to informed consent, as the UK Supreme Court did in *Montgomery*, is an interesting development. The purpose of this article is to explore this comparison, in the light of the growing body of contract and consumer law scholarship which questions the extent to which information disclosures to consumers produce informed choices.

As is well known, following the UK Supreme Court’s decision in *Montgomery*, it is not enough for doctors to provide generic information about a treatment’s risks. Instead, before giving consent to medical treatment, a patient should be told about a risk if ‘the doctor is or should reasonably be aware that the particular patient would be likely to attach significance to it’.^3^ In order to find out what matters to the individual patient, doctors must now engage in the sort of dialogue which Lords Kerr and Reed noted has ‘long’ been a requirement of General Medical Council (GMC) guidance.^4^ Because the standard of care in tort law is ‘now consistent with the GMC professional guidance’,^5^ there have been suggestions that the UK Supreme Court’s ‘landmark’^6^ decision in *Montgomery* may not make much difference in practice.^7^

In this journal, Arvind and McMahon have drawn attention to one way in which the judgment in *Montgomery*, in fact, diverges from the GMC’s partnership model of decision making.^8^ Lords Kerr and Reed pointed to the increasing tendency to regard patients as rights holders, and ‘as consumers exercising choices’,^9^ to whom what looks like the principle of *caveat emptor* might sometimes apply:

The social and legal developments which we have mentioned point away from a model of the relationship between the doctor and the patient based on medical paternalism. They also point away from a model based on a view of the patient as being entirely dependent on information provided by the doctor. What they point towards is an approach to the law which, instead of treating patients as placing themselves in the hands of their doctors (and then being prone to sue their doctors in the event of a disappointing outcome), treats them so far as possible as adults who are capable of understanding that medical treatment is uncertain of success and may involve risks, accepting responsibility for the taking of risks affecting their own lives, and living with the consequences of their choices.^10^

As Purshouse has observed, by characterising patients ‘as capable adults responsible for their own choice’, informed consent cases appear ‘to be developing separate rules to those governing the rest of medical negligence’,^11^ where judges continue to draw attention to patients’ vulnerability.^12^ For the UK Supreme Court to draw an analogy between patients and consumers in the context of informed consent is also interesting given the increasing recognition that consumers fail routinely to understand and use information disclosures.

Behavioural economics has had a significant impact upon contract and consumer law scholarship because it raises questions about the way in which human beings use information in order to make decisions. Is informed consent to medical treatment an entirely different matter, or might it be possible to compare patients with consumers, not because they ‘are now widely regarded as persons holding rights’,^13^ but rather because, like consumers, they might not always understand the information they have received, or use it in order to make an informed choice? If there is evidence that the duties of disclosure which are imposed on retailers, in order to redress the imbalance of knowledge and power in the consumer–retailer relationship,^14^ do not always fulfil their intended purpose, might this have any resonance for the duties of disclosure which are imposed upon healthcare professionals?

Of course, there are multiple and significant differences between information disclosures which are made before a consumer enters into a contract with a retailer, and the information patients receive before they give consent to medical treatment. Consenting to surgery is indubitably not like buying a new phone. Doctors (and other healthcare professionals) owe professional obligations towards their patients which are unlike retailers’ contractual duties towards their customers. Information disclosures to consumers are also standardised, whereas doctors are under a duty to tailor information to the needs of the individual patient.

Nevertheless, given that the Supreme Court has drawn an analogy between patients and consumers in the context of information disclosure, it is worth drawing attention to evidence that disclosures which look like they should help consumers make better decisions do not work, because they rest ‘on false assumptions about how people live, think, and make decisions’.^15^ Are these ‘false assumptions’ about how people use information in order to make decisions applicable only when someone is buying a product or service, or might they also affect how people use information in order to make medical decisions? Or, to put it another way, if people struggle to digest and use information given to them when they are consumers, is it at least plausible that the same people might also struggle to digest and use information when they are presented with it as patients?

Although the jumping-off point for this article is the analogy the UK Supreme Court drew between patients and consumers in *Montgomery*, this article will first set out the many ways in which consumers and patients are differently situated in relation to information disclosures. Secondly, I turn to consider how, relying upon insights from behavioural economics, contract and consumer law scholars have demonstrated that pre-contractual disclosures routinely fail to produce informed decisions. While patients generally receive more targeted and trustworthy information than consumers, it is not clear that this will eradicate some of the inherent difficulties people face when trying to understand and use information. Thirdly, I will acknowledge that it would be a mistake to lump all patients together, and that, in practice, patients’ appetite for information varies, as does the value they attach to it,^16^ and their capacity to use it in order to make decisions. Finally, the point of the comparison Lords Kerr and Reed drew between patients and consumers in *Montgomery* was in order to emphasise the priority now given to patient autonomy. In what follows, I suggest that this comparison could usefully serve an altogether different purpose, namely, to focus our attention upon how challenging it can be to communicate effectively with patients.

## II. DIFFERENCES BETWEEN CONTRACTUAL AND MEDICAL DISCLOSURES

There are multiple differences between pre-contractual information disclosures to consumers and the process of gaining a patient’s informed consent to medical treatment. First, as Tallis explained in the context of a debate over whether patients should be redefined as customers:

Someone who is ill and seeking help—unlike someone who is purchasing a pair of socks or a pound of sausages—is often vulnerable, certainly worried, sometimes uncomfortable, and frequently frightened. Customer, like the other obvious choices—clients, consumers, and users—erases something that lies at the heart of medicine: compassion and a relationship of trust.^17^

The relationship a consumer has with a retailer will almost always be less intimate and trusting than the doctor–patient relationship. Not only are doctors in a relationship of trust with their patients, they are also under a professional duty to make the care of their patient their first concern.^18^ It is unusual for a retailer to be under a duty to question a consumer’s choices,^19^ whereas a healthcare professional should query a patient’s decision, if it seems to be at odds with her previously expressed wishes.^20^ In *Mordel v Royal Berkshire NHS Foundation Trust*,^21^ for example, Ms Mordel had initially decided to undergo all six standard prenatal screening tests, but then answered ‘no’ when asked whether she wanted the test for Down’s syndrome. Jay J found that the sonographer had been negligent in failing to question whether Ms Mordel, whose English was not perfect, had understood the question, given that it contradicted her earlier choice.

Medical decisions may also be more difficult than the decision to buy a new product, involving, as Epstein puts it, ‘a series of complex trade-offs’:

Should a patient choose a riskier procedure that may lead to the best possible outcome but with the greatest potential side effects, or the safer option where the patient knows the outcome will not be ideal? How should a patient choose between the psychological burden of a wait-and-see approach versus the potential complications and cost of a serious surgery? How does a patient weigh the cost in taking extended time away from work for recovery against a longer-term reduction in ability from not acting?^22^

In addition to considering their own wellbeing, patients are often concerned about the impact of their condition and its treatment upon their dependants.^23^ Patients are therefore making multi-faceted and challenging decisions, from a position of vulnerability, and in the context of a doctor–patient relationship where there may be a high level of trust, and even dependence. Indeed, this is why, as Purshouse has explained, invoking the metaphor of ‘patients as consumers’ in an informed consent case represents a departure from the more usual depiction of patients in clinical negligence cases as potentially, if not inherently vulnerable.^24^

Secondly, as we see later, mandatory disclosures are a low-cost consumer protection technique precisely because they are standardised.^25^ Warning all consumers about a risk is simpler and cheaper than either removing the risk, or targeting the disclosure at particular consumers. In contrast to this blanket approach, the information that patients receive before giving informed consent to medical treatment should be adapted in order to meet the specific needs of the individual patient. Patient information leaflets are insufficient on their own, and must be accompanied by dialogue, in which the doctor tries to find out what matters to this patient. The GMC’s latest good practice guidance on consent instructs doctors to ‘explore your patient’s needs, values and priorities that influence their decision making, their concerns and preferences about the options and their expectations about what treatment or care could achieve’,^26^ suggesting that doctors ‘should ask questions to encourage patients to express what matters to them, so you can identify what information about the options might influence their choice’.^27^

Dialogue with patients costs money, most significantly in staff time,^28^ so a high-quality informed consent process will be anything but cheap. If the patient needs to reflect upon the information she has received, extra appointments may be necessary. In *Montgomery*, Lords Kerr and Reed acknowledged that the obligation ‘to pause and engage in the discussion which the law requires … may not be welcomed by some healthcare providers’.^29^ There is, in fact, some evidence that since *Montgomery*, ‘pre-treatment counselling’ is now taking longer, and hence the costs of obtaining informed consent have risen.^30^ The good practice guidance published by the Royal College of Surgeons in response to *Montgomery* warns that ‘[t]he reality facing surgeons in current practice is that time pressures can leave little opportunity to discuss at length the diagnoses or available treatment options’.^31^ A survey of doctors commissioned by the GMC, prior to the 2020 revisions to its consent guidance, found that ‘doctors, working in many different specialisms, reported that individualising the consent conversation takes time that they (and the NHS) cannot afford’ .^32^

Thirdly, the purpose of pre-contractual disclosures may be different from medical disclosures in two important ways. First, although, as Howells points out, ‘regulatory policy is no longer fixated with the idea of a malevolent trader trying to con consumers but, rather, focuses on the asymmetries of information between trader and consumer’,^33^ retailers are nevertheless trying to sell their products and services to consumers. If retailers are under an obligation to disclose certain information to potential consumers, they may deliberately include it in their ‘small print’ terms and conditions, which they know are seldom read.^34^

In contrast, doctors do not generally take advantage of their patients’ unwillingness to read terms and conditions in order to sell them treatments that are inappropriate for them. There may be some atypical pockets of private practice where there is a risk of overselling,^35^ but a doctor would be putting her registration at risk if she took advantage of patients’ inability to understand disclosures in order to encourage them to make choices that were not in their best interests. In *Plant v El-Amir*, for example, Stacey J noted that Dr Qureshi, who had misled the claimant about the risks and benefits of expensive, experimental eye surgery, ‘has had his name erased from the GMC and been struck off for putting his financial interests above those of his patients’.^36^

Secondly, pre-contractual disclosures are often explicitly intended to reduce or eliminate liability. Even if there is evidence that some patients think that by signing a consent form, they have waived their right to sue if something goes wrong,^37^ the consent form is not a contract, and the patient is free to change her mind after signing it.

As a result of these multiple differences, there are good reasons to be cautious about drawing too close an analogy between patients and consumers in the context of information disclosure. Of course, the people who may struggle to understand and use the disclosures that they receive as consumers are the same people who receive information from healthcare professionals when they are patients. But, as patients, the information they receive will be more tailored to their needs than the information they receive as consumers, and they will have an opportunity to talk to a specialist, who is furthermore obliged to make their care her first concern. Patients giving informed consent to medical treatment may therefore receive more useful information than consumers, and they will be able to place more trust in the information provider. As we see in the following section, however, contract and consumer law scholars do not tend to attribute consumers’ failure to understand and use information disclosures to the fact that information is either standardised or comes from an untrustworthy source.

## III. INFORMATION DISCLOSURES TO CONSUMERS

It is true, as Arvind and McMahon have pointed out, that few patients correspond to ‘the ideal type of the self-aware, informed, perfectly confident patient-consumer’.^38^ It is, however, also important to recognise that most *consumers* do not correspond to the ‘the ideal type of the self-aware, informed, perfectly confident’ contracting party. A consumer who wants to purchase a new phone does not bargain at arms-length with Apple or Samsung over the terms upon which she is willing to enter into a contract with them. Consumers exercise no control over the terms of ‘boilerplate’ contracts, other than not to enter into them. If they want to buy something, they must agree to lengthy terms and conditions which are often intended to protect the interests of the more powerful party.^39^

As Howells has pointed out ‘the provision of information is one of the key tools available to enhance consumer protection’.^40^ According to classical contract theory, information provision serves multiple desirable goals:

Briefly stated, consumers have less information than traders and so have difficulty in making decisions that reflect their true preferences. There are not sufficient incentives for traders to volunteer information, so the law needs to require that the information be provided. Once this information is provided, consumers can protect their own interests by selecting the goods or services closest to their preferences. Harm will be reduced by ensuring goods and services are more likely to be in line with realistic consumer expectations based on reliable information. Avoiding problems through the consumer taking responsibility for his or her own purchasing choices must be a desirable objective.^41^

Because ‘the adoption of information obligations for traders is a measure that intrudes upon the freedom of the marketplace and party autonomy only to a small extent’,^42^ ‘“empowering” consumers through information has become a singularly important element in the regulatory toolbox’.^43^

The idea that disclosure obligations enhance both autonomy and consumer protection, at relatively low cost, has led to their proliferation. The assumption is, as Sibony and Helleringer put it, that ‘more information is always better for consumers’.^44^ Over the past two decades, contract and consumer law theorists have increasingly questioned this orthodoxy. They have done so by drawing upon evidence from behavioural economists and applied psychologists which demonstrates that, while there is considerable heterogeneity in our appetite for information,^45^ it is in fact normal for people routinely to avoid or misunderstand disclosures.^46^ According to Sibony and Helleringer:

The behavioral critique strikes EU consumer law at its heart, by questioning its preferred regulatory approach: the information paradigm, which has characterized EU consumer law since it came into existence.^47^

Crucial to this ‘behavioural critique’ is the recognition that the failure to understand disclosures is not, as is sometimes assumed, confined to people who are especially vulnerable. Even if information disclosures are more likely to be used by ‘the more affluent, well-educated middle-class consumers’,^48^ the tendency to misunderstand disclosures and misread information, is certainly ‘not limited to the uneducated and unintelligent’.^49^

Using information disclosure as a consumer protection technique rests on the assumption that the average consumer has the capacity to process information and act on it, and that it is only atypical vulnerable consumers who need special protection. Yet, as Howells points out, ‘The truth is that we are all to some extent vulnerable, because of the limitations of the human mind.’^50^ Oren-Gill and Ben-Shahar explain that:

Extensive past experience in consumer protection suggests that standard consumer ‘informed consent’ techniques fail. They are not read nor used, and they are beyond most people’s care or understanding… People do not pay attention to standard forms, neither long nor short, in plain language or in legalese, written or oral, separately signed or unified into one document, handed out in advance or ex post.^51^

According to Loewenstein and others, and with obvious resonance for patients as well as consumers, there are ‘serious limitations on the amount of information to which people can attend at any point in time. Bounded attention renders many disclosures useless because consumers ignore them.’^52^ Too much information can be as much of an obstacle to informed choice as too little.^53^

When information is complex, consumers may deal with that complexity by ignoring it.^54^ Information can also have an opportunity cost^55^; in addition to being difficult to digest, some information is alarming, and consumers may prefer not to know about it.^56^ Furthermore, the widespread assumption that consumers know better than sellers how they are likely to use a product is also false^57^: consumer estimates of use tend to be wildly inaccurate, while many retailers possess sufficient data to be able to make precise predictions about consumer behaviour.^58^

Behavioural economists have additionally pointed out that when they interpret information about risk, it is normal for people to be over-optimistic and over-confident. Over-optimism, as Williams explains:

causes people to underestimate their likelihood of negative events, to overestimate the likelihood of positive events, and to be overly confident in each of these erroneous judgments. It also distorts people’s reactions to explicit and accurate probabilistic information.^59^

Optimism bias is not universal: Sharot has pointed out that ‘people with mild depression show no bias when predicting future events, and people with severe depression tend to expect things to be worse than they turn out’.^60^ This, according to Sharot, leaves approximately 80% of the population who ‘expect the future to be slightly better than it ends up being’, a tendency which she describes as ‘one of the most consistent, prevalent, and robust biases documented in psychology and behavioral economics’.^61^

If told that a website has a privacy policy, for example, many consumers assume that this protects their privacy, rather than facilitating the wider use of their data.^62^ It is common for people to update their beliefs more frequently in response to good news than they do when the news is less favourable.^63^ There is also evidence that recall of information can be selective. People may be more likely to remember being told about benefits than risks, and they may discount information about average risks because of their over-optimistic belief that they are less likely to suffer negative outcomes than the average person.^64^ People often interpret information in a way that bolsters and supports their preconceptions, so that they believe information which confirms their prior view and discredit information that does not.^65^ Statistical evidence may be discounted in favour of anecdotal experience, and initial judgements can be hard to displace.^66^

Very few consumers read contractual terms and conditions before they enter into a contract, and they are certainly not the principal source of information for consumers contemplating purchasing a product or service. As Seizov and others put it in the context of online consumer behaviour: ‘The omnipresence of disclosures in modern internet users’ daily lives is rivalled only by said users’ distaste for acknowledging and reading them.’^67^

Instead, consumers are generally more interested in the ratings and endorsements of others, both about the usefulness of the product and the trustworthiness of its seller, than they are in the ‘mandated disclosures’ which they receive before entering into a contract. Ben-Shahar and Schneider explain that: ‘Faced with unfamiliar and complex decisions that disclosures are intended to inform, people don’t want to be educated, don’t want spreadsheets and don’t want scrolls. *They want advice*’.^68^ Of course, as Ezechukwu points out, consumers’ understanding of advice, which frequently takes the form of consumer-generated reviews (CGRs), is not immune to the cognitive biases and other ‘limitations of human information processing’ which interfere with their capacity to understand mandatory disclosures: there is, for example, some evidence that consumers latch ‘onto particular CGRs which confirm their preconceived notions of a product’.^69^

As we see in the next section, patients might also struggle to understand information about risk and value advice, but it is important to reiterate that undergoing medical treatment is radically different from shopping. Doctors’ obligations towards their patients are clearly more stringent and demanding than those imposed on retailers. Nevertheless, the evidence is not that consumers struggle to understand and use information disclosures because they are standardised, or buried in small print. Rather, the reason why contract and consumer lawyers have drawn on behavioural economics and applied psychology is in order to highlight the *inherent* challenges human beings face in digesting information and using it in order to make informed choices. These challenges exist not because of the specific content of the information, or because of the nature of the relationship between the person disclosing the information and the person receiving it. Rather it is the way in which human beings reason and make decisions that is at issue. If over-optimism is a ‘consistent, prevalent, and robust’ bias, its influence is unlikely to be confined only to purchasing decisions, but might also extend to how someone understands the risks of medical treatment, or the chances of its success.

## IV. PATIENTS’ USE OF INFORMATION DISCLOSURES

What we might describe as the *Montgomery* model of informed consent assumes that, following information disclosure and discussions with their doctor, patients will understand the risks associated with treatment, and any alternatives to it, and will decide whether to proceed after having considered this information, in the light of their values.^70^ In practice, however, there is evidence that medical decisions are not always made in this rational, linear way. Patients may not understand what they have been told, and the information they receive may have little impact upon their choices. This should not be surprising. If psychologists and behavioural economists are right about the limitations on human beings’ reasoning capacities and their decision-making biases, it would be peculiar if these had no impact at all in other decision-making contexts.

It is, of course, important to recognise that patients’ appetite for information, and their understanding of it varies considerably. Not only are there differences between patients, but the same patient’s preferences may vary over the course of her lifetime, and in response to different illnesses. Patients who suffer from chronic conditions may ‘become experts in their own conditions and sometimes know more than generalists (such as their GPs) about symptoms and management options’.^71^ At the same time, as Arvind and McMahon point out, there is evidence from patient survey data that other patients may struggle to understand or retain the information that they receive as part of the informed consent process.^72^ Information about risk is notoriously difficult to understand,^73^ and the way in which information is framed can be critical: if patients are told that 90% of people are alive five years after having an operation, they are more likely to consent than if they are told that 10% are dead.^74^

When explaining the ‘material risks’ of a procedure, doctors often rely on ‘objective probability estimates’, which are ‘derived from and expressed in terms of the observed frequency of past outcomes in a population of individuals, and enable inferences about the frequency of expected future outcomes in a similar population’.^75^ But while estimates of population-wide risk make sense at a macro level, for the individual patient, whether or not she will experience a rare side effect is, in fact, binary: it will either happen, or it (more likely) will not happen. Han has therefore pointed out that ‘objective probability estimates are logically incoherent when applied to the prospect of a future event experienced by a single individual with only one life to live’.^76^ On the one hand, in order to be strictly accurate, objective probability estimates should perhaps come with a ‘health warning’ about their application to the individual patient. On the other hand, this is likely to make information even less digestible. For example, as Han explains,

A woman who learns … that her lifetime risk of breast cancer is ‘12%’ … needs to be made aware that ‘12%’ is not a literal representation of her own ‘true’ risk but a figurative expression of scientists’ *confidence* based on the aggregated outcomes of individuals whose characteristics are similar—but not completely equivalent—to her own.^77^

Healthcare professionals may face particular difficulties in communicating about uncertainty and the risk of failure.^78^ The tendency discussed earlier for people to be over-optimistic when presented with information about risk has also been studied in the medical context. When doctors express prognostic uncertainty, it is common for patients to ‘mistakenly place themselves in the most optimistic prognostic group’^79^; or to ‘view hazards as more risky for other people than for themselves’.^80^ Where a treatment has a low chance of success, it is not necessarily sufficient for a doctor simply to alert the patient to this. Optimism bias and the fact that the doctor is willing to proceed with treatment in their case, may encourage patients to believe that *their* treatment is likely to succeed.^81^

When healthcare professionals tell patients that their risk of chronic pain is ‘small’,^82^ or that their condition is ‘treatable’, they should not assume that their patients’ understanding of these words is the same as theirs. To a healthcare professional, ‘treatable’ may simply mean that there is some treatment available, while patients may hear that their condition can be cured. As Batten and others explain:

nonphysicians hear the word ‘treatable’ as conveying good news about the future, thereby inspiring hope and encouraging further treatment. In contrast, physicians use the word ‘treatable’ in a technical sense, to convey that they have an action or intervention available, which does not necessarily imply an improved prognosis or quality of life.^83^

Identifying and trying to correct this sort of misunderstanding should be as routine and essential a component of the informed consent process as the disclosure of material risks and alternatives.^84^

Patients’ ‘pathologies of reasoning’^85^ are not confined to over-optimism. As King and Moulton explain, patient comprehension may also be affected by ‘availability bias’, where patients ‘overestimate their risk of contracting a condition that receives substantial media coverage, such as breast cancer’; ‘compression bias’, which involves ‘patients overestimating small risks and underestimating large ones’; ‘small numbers bias’ where patients ‘misinterpret their individual risk based on a small number of known cases (my two friends both had complications after their hysterectomies, so I probably will too)’; and ‘miscalibration bias’ which involves patients being ‘overly confident about the extent or accuracy of their knowledge’.^86^

In addition, patients do not necessarily take medical decisions *after* having received information about a treatment’s risks and benefits, which they have weighed up in the light of their values. In practice, it is not uncommon for patients to have already made the decision to be treated before they encounter the doctor who is responsible for telling them about the treatment’s risks and benefits.^87^ Some patients base their decisions on intuition or instinct,^88^ or ‘seize on a single aspect of a problem and make it the basis for their choice’, and once they have done that, ‘any new evidence is taken to confirm that choice’.^89^ When facing a cancer diagnosis, for example, patients commonly express a preference for invasive surgery ‘even when they would be better off doing nothing’, because of their ‘intuitive belief that cancers should not simply be treated but should be removed’.^90^ Sauder and Parker’s research with individuals who had donated a kidney to their child or sibling found that most ‘experience the decision to donate as automatic’.^91^

If some patients have already taken the decision to proceed before they hear about the treatment’s risks and benefits, the informed consent process can become a bureaucratic ‘tick-box’ exercise which must be ‘gone through’ in order that treatment can take place.^92^ In the case of surgery and other invasive procedures, this may be exacerbated by the use of consent forms which are widely perceived to be ‘a purposeless bureaucratic or legalistic ritual that ostensibly protects doctors and not their patients’.^93^ Just like consumers who do not read terms and conditions before ticking the box to confirm that they have read them, it is not uncommon for patients to skim read consent forms, or not to read them at all.^94^ The problem is not just patient inattention; because information leaflets and consent forms assume a level of literacy that is lacking in a substantial proportion of the population, for many patients, they are simply incomprehensible.^95^

There are also cultural and other variations in how much information patients want, and in whether they want to make their own medical decisions.^96^ Not all patients will conform to the paradigm of the autonomous patient, who wants to be told what their prognosis is, and what treatments are available, with what risks and benefits, before making a decision on their own. On the contrary, this may be a culturally specific preference.^97^ Among some minority ethnic groups, there is evidence that it is more common for patients not to want to know if their prognosis is poor, or to want their family, or their doctor, to make decisions for them.^98^ Disclosures are not necessarily empowering for these patients, rather they may be perceived as burdensome and even ‘cruel’.^99^

In addition to variation in patients’ appetite for and use of information, there is also variation in how much value they attach to information disclosures. Arvind and McMahon have pointed to evidence that what some patients value most in their pre-treatment encounters with doctors is not necessarily information about risks and alternatives^100^; instead, patients might be more concerned about whether their health concerns are being taken seriously, and whether they are treated with compassion and respect.^101^ Surveys of what matters most to patients often rank being able to trust healthcare professionals above the right to make autonomous decisions.^102^ Interpersonal skills are important: patients want their doctors to be sensitive to the embarrassment of being naked in front of fully dressed professionals, to introduce themselves, and not to speak to colleagues over them, as if they are not there.^103^

It could, of course, be argued that providing patients with information in order to facilitate decision making is a way of treating a patient as worthy of respect. Manson has, for example, suggested that:

The fact that a clinician is willing to inform a patient about treatment options may be viewed as indicative of respect (provided it is done so in respectful manner). The clinician treats the patient as someone who is capable of being informed, and who has an interest in being informed. A patient can have an interest in being respected without thereby wanting to make decisions herself. The fact that the clinician is willing to engage in communication may help to inspire confidence in the clinician as a trustworthy agent.^104^

In a similar vein, O’Neill has argued that the purpose of informed consent is not so much to ensure patients make autonomous choices, but rather to guard against deception and coercion.^105^

Even for patients who are ‘decision averse’,^106^ pre-treatment discussions may nevertheless serve several important purposes. Talking to a patient about her condition and its treatment can—if done well—be a way of demonstrating that she is being taken seriously. Information can also be reassuring and help patients to prepare themselves for what is going to happen. As Manson further explains:

It is not irrational, or irrelevant to seek assurances that the decision will be made in a reasonable way, and the disclosure of information can provide this kind of assurance. That is, a patient can want to be assured that a good decision will be made (by someone else) without wanting to make that decision herself.^107^

Acknowledging human frailties in comprehension and decision making does not therefore amount to an argument against the provision of information to patients before they consent to medical treatment. Rather, my claim is that a good informed consent process should not assume that the neutral presentation of information about material risks and alternatives will seamlessly produce informed patients making informed decisions.

## VI. CONCLUSION

Patient autonomy has become the dominant value of late twentieth and early twenty-first century bioethics and of medical law, and the UK Supreme Court judgment in *Montgomery* has been hailed as another milestone in autonomy’s triumph over paternalism. While I am not advocating a return to paternalism, it is important to acknowledge that the circumstances in which medical decisions have to be made are often less than ideal, involving stress, anxiety, and feelings of vulnerability and dependency.^108^ In practice, consenting patients may not have much in common with ‘the rational autonomous actor, so beloved of bioethics’, who makes calm, well-ordered decisions on the basis of objective medical facts.^109^

The doctrine of informed consent is intended to buttress patient autonomy by equipping patients with the information they need in order to exercise freedom of choice over the medical treatment they receive. Medical decisions are often unfamiliar and complex, and disclosure offers an ‘alluringly simple’ solution: ‘give [people] information until the decision is familiar and comprehensible’.^110^ But, as Levy has explained, while it may have been ‘laudable’ to make the ‘doctrine of informed consent’ central to medical ethics, ‘it rests on implicit assumptions with regard to the capacities of normal human beings that may be unrealistic’.^111^ Levy goes on to claim that, insofar as the doctrine of informed consent depends upon the ‘supposition that normal human beings can be expected, unaided and in stressful and novel contexts, to make choices that contribute to the achievement of their own most cherished ends, it seems to be in trouble’.^112^

If there is an interesting point of comparison between patients and consumers, it is not that patients are ‘widely treated as consumers exercising choices’, for which they should accept responsibility. Instead, it could be argued that the duties of disclosure which are imposed on doctors (and retailers), in order to redress imbalances of knowledge and power in the doctor–patient (and consumer–retailer) relationship,^113^ are not always necessarily understood or used in order to make informed decisions. This is not to argue that we should abandon the requirement that doctors share relevant and comprehensible information with patients before they consent to medical treatment. Rather, my claim is that we should take seriously the idea that information disclosures are not always easy to understand, and that they may not be used by patients in order to make informed choices.

In *Montgomery*, Lords Kerr and Reed invoked the metaphor of ‘patients as consumers’ in order to emphasise the importance of patients’ right to make informed choices about their medical treatment. This is, I have argued, a striking analogy in the light of recent consumer and contract law scholarship which demonstrates that information disclosures to consumers do not result in informed consumer decisions. Importantly, disclosures to consumers fail not because they are standardised and designed to protect retailers’ interests—which would clearly distinguish them from medical disclosures—but because of the array of biases and limitations which shape human beings’ capacity to understand and use information. A comparison between patients and consumers could, therefore, lead in a rather different direction from that intended by Lords Kerr and Reed, namely, towards a deeper appreciation of how challenging it can be to understand information disclosures and to use them in order to make informed choices.

## Footnotes

1 [2015] UKSC 11. In brief, the facts in *Montgomery* were that Ms Montgomery was not informed about the risk of shoulder dystocia, associated with the vaginal delivery of a larger than average baby. If she had been informed, she would have insisted upon a caesarean delivery, and her son would not have suffered the injuries he sustained during childbirth as a result of mechanical efforts to deal with his shoulder dystocia.

2 See eg J Tritter and others, *Globalisation, Markets and Healthcare Policy: Redrawing the Patient as Consumer* (Routledge 2009).

3 Per Lords Kerr and Reed, *Montgomery* (n 1) [87].

4 ibid [93]. More than two decades ago, the GMC’s 1998 consent guidance instructed doctors that ‘[w]hen providing information you must do your best to find out about patients’ individual needs and priorities’ *Seeking Patients’ Consent: The Ethical Considerations* (GMC 1998), para 6. According to the fourth principle of its 2020 guidance, ‘doctors must try to find out what matters to patients so that they can share relevant information about the benefits and harms of proposed options and reasonable alternatives’ *Decision Making and Consent* (GMC 2020).

5 L Sutherland, ‘Montgomery: Myths, Misconceptions and Misunderstanding’ (2019) 3 Journal of Personal Injury Law 157–67.

6 S Chan and others, ‘*Montgomery* and Informed Consent: Where Are We now?’ (2017) 357 British Medical Journal j2224; JV McHale, ‘Innovation, Informed Consent, Health Research and the Supreme Court: *Montgomery v Lanarkshire*–A Brave New World?’ (2017) 12 Health Economics, Policy and Law 435–52; M Robson and K Swift, ‘Risk Disclosure, Causation and the Role of *Chester*’ (2019) 35 Professional Negligence 207–29.

7 See eg R Heywood, ‘RIP Sidaway: Patient-Oriented Disclosure—A Standard Worth Waiting For?’ (2015) 23 Medical Law Review 455–66; AM Farrell and M Brazier ‘Not So New Directions in the Law of Consent? Examining *Montgomery v Lanarkshire Health Board*’ (2016) 42 Journal of Medical Ethics 85–88; J Herring and others, ‘Elbow Room for Best Practice? *Montgomery*, Patients’ Values, and Balanced Decision-Making in Person-Centred Clinical Care’ (2017) 25 Medical Law Review 582–603.

8 TT Arvind and AM McMahon, ‘Responsiveness and the Role of Rights in Medical Law: Lessons from *Montgomery*’ (2020) 28 Medical Law Review 445–77.

9
*Montgomery* (n 1) [75]

10 ibid [75] and [81].

11 C Purshouse, ‘The Impatient Patient and the Unreceptive Receptionist: *Darnley v Croydon Health Services NHS Trust*’ (2019) 27 Medical Law Review 318–29.

12 See eg Lord Lloyd Jones in *Darnley v Croydon Health Services NHS Trust* [2018] UKSC 50 at [29]; Lord Sumption in *Woodland v Essex County Council* [2013] UKSC 66 at [23]; and Lord Dyson in *Farraj v King’s Healthcare NHS Trust* [2009] EWCA Civ 1203 [88].

13
*Mongtomery* (n 1) [75]

14 O Bar-Gill, ‘Consumer Transactions’ in E Zamir and D Teichman (eds), *The Oxford Handbook of Behavioral Economics and the Law* (OUP 2014) 465–90.

15 CE Schneider and O Ben‐Shahar, ‘The Failure of Mandated Disclosure’ (2010) 159 University of Pennsylvania Law Review 659

16 N Hallowell, ‘Encounters with Medical Professionals: A Crisis of Trust or Matter of Respect?’ (2008) 11 Medicine, Health Care and Philosophy 427–37.

17 R Tallis, ‘Commentary: Leave Well Alone’ (1999) 318 British Medical Journal 1756–58.

18
*Good Medical Practice* (GMC 2019).

19 An exception might be affordability requirements for mortgages and other types of credit.

20 J Wilson, ‘Is Respect for Autonomy Defensible?’ (2007) 33 Journal of Medical Ethics 353–56.

21 [2019] EWHC 2591

22 WN Epstein, ‘Nudging Patient Decision-Making’ (2017) 92 Washington Law Review 1255.

23 A Lippman, ‘Choice as a Risk to Women’s Health’ (1999) 1 Health, Risk & Society 281–91. N Stoljar, ‘Informed Consent and Relational Conceptions of Autonomy’ (2011) 36 Journal of Medicine and Philosophy 375–84

24 Purshouse (n 11).

25 G Howells, ‘The Potential and Limits of Consumer Empowerment by Information’ (2005) 32 Journal of Law and Society 349–70.

26 GMC (2020) (n 4) para 18.

27 ibid, para 19.

28 PH Schuck, ‘Rethinking Informed Consent’ (1994) Yale Law Journal 899–959.

29
*Montgomery* (n 1), *per* Lords Kerr and Reed at [90].

30 N Harrison and others, ‘How *Montgomery* is Reconfiguring Consent in the UK’ (2018) 392 The Lancet 102–4; S Devaney and others, ‘The Far-Reaching Implications of *Montgomery* for Risk Disclosure in Practice’ (2019) 24 Journal of Patient Safety and Risk Management 25–29.

31 Royal College of Surgeons, *Consent: Supported Decision-Making - A Good Practice Guide* (RCS 2016)

32 Community Vision, *Doctors' Attitudes to Consent and Shared Decision Making: Full Research Report for the* *GMC* (GMC, 2017) 27.

33 Howells (n 25).

34 Bar-Gill (n 14); X Gabaix and D Laibson, ‘Shrouded Attributes, Consumer Myopia, and Information Suppression in Competitive Markets’ (2006) 121 Quarterly Journal of Economics 505–40. O Bar-Gill, *Seduction by Contract: Law, Economics, and Psychology in Consumer Markets* (OUP 2012).

35 Infertility treatment is one example, and has recently been the subject of two sets of guidance to patients and to clinics from the Consumer and Markets Authority: Consumer and Markets Authority, *Fertility Treatment: A Guide to Your Consumer Rights* (CMA 2021) and *Fertility Treatment: A Guide for Clinics: Helping Fertility Clinics Comply with their Consumer Law Obligations* (CMA 2021). See also J Wilkinson and others, ‘Do à La Carte Menus Serve Infertility Patients? The Ethics and Regulation of IVF Add-ons’ (2019) 112 Fertility and Sterility 973–77.

36 [2020] EWHC 2902 (QB) [25].

37 A Akkad and others, ‘Patients’ Perceptions of Written Consent: Questionnaire Study’ (2006) 333 British Medical Journal 528; M Habiba and others, ‘Women’s Accounts of Consenting to Surgery: Is Consent a Quality Problem?’ (2004) 13 Quality and Safety in Health Care 422–27; N Eyal, ‘Using Informed Consent to Save Trust’ (2014) 40 Journal of Medical Ethics 437–44.

38 Arvind and McMahon (n 8).

39 C Leonhard, ‘The Unbearable Lightness of Consent in Contract Law’ (2012) 63 Case Western Reserve Law Review 57; MJ Radin, *Boilerplate: The Fine Print, Vanishing Rights, and the Rule of Law* (Princeton University Press 2013).

40 Howells (n 25).

41 ibid.

42 S Grundmann, ‘Information, Party Autonomy and Economic Agents in European Contract Law’ (2002) 39 Common Market Law Review 269–93.

43 N Helberger, ‘Diversity label: Exploring the Potential and Limits of a Transparency Approach to Media Diversity’ (2011) 1 Journal of Information Policy 337–69.

44 AL Sibony and G Helleringer, ‘European Consumer Protection through the Behavioural Lens’ (2017) 23 Columbia Journal of European Law 607–46.

45 CR Sunstein, ‘Ruining Popcorn? The Welfare Effects of Information’ (2019) 58 Journal of Risk and Uncertainty 121–42.

46 Y Bakos, F Marotta‐Wurgler and DR Trossen, ‘Does Anyone Read the Fine Print? Testing a Law and Economics Approach to Standard Form Contracts’ (2009) 9 New York University Law and Economics Research Paper Series 40; R Hertwig and C Engel, ‘Homo Ignorans: Deliberately Choosing Not to Know’ (2016) 11 Psychological Science 359–72; O Bar-Gill, D Schkade and CR Sunstein, ‘Drawing False Inferences from Mandated Disclosures’ (2019) 3 Behavioural Public Policy 209–27.

47 Sibony and Helleringer (n 44).

48 Howells (n 25)

49 ibid.

50 ibid.

51 O Bar-Gill and O Ben-Shahar, ‘Regulatory Techniques in Consumer Protection: A Critique of the Common European Sales Law’ (2012) 12 New York University Law and Economics Research Paper Series 1–33.

52 G Loewenstein, CR Sunstein and R Golman, ‘Disclosure: Psychology Changes Everything’ (2014) 6 Annual Review of Economics 391–419.

53 KB Schulz, ‘Information Flooding’ (2014) 48 Indiana Law Review 755.

54 O Bar-Gill, ‘Competition and Consumer Protection: A Behavioral Economics Account’ in Konkurrensverket Swedish Competition Authority, *The Pros and Cons of Consumer Protection* (Konkurrensverket 2011) 11–42.

55 Loewenstein, Sunstein and Golman (n 52).

56 Sunstein (n 45).

57 A Schwartz and LL Wilde, ‘Intervening in Markets on the Basis of Imperfect Information: A Legal and Economic Analysis’ (1979) 127 University of Pennsylvania Law Review 630.

58 O Bar-Gill and O Board, ‘Product-Use Information and the Limits of Voluntary Disclosure’ (2012) 14 American Law and Economics Review 235–70.

59 SH Williams, ‘Probability Errors: Overoptimism, Ambiguity Aversion, and the Certainty Effect’ in Zamir and Teichman (n 14) 335–53.

60 T Sharot, ‘The Optimism Bias’ (2012) 21 Current Biology R941–45. See also DR Strunk, H Lopez and RJ DeRubeis, ‘Depressive Symptoms Are Associated with Unrealistic Negative Predictions of Future Life Events’ (2006) 44 Behaviour Research and Therapy 861–82.

61 Sharot, ibid. See also T Sharot, *The Optimism Bias: Why We’re Wired to Look on the Bright Side* (Hachette 2012).

62 J Turow and others, ‘The Federal Trade Commission and Consumer Privacy in the Coming Decade’ (2007) 3 I/S: A Journal of Law and Policy for the Information Society 723–49.

63 T Sharot and others, ‘Selectively Altering Belief Formation in the Human Brain’ (2012) 109 Proceedings of the National Academy of Sciences of the United States of America 17058–62.

64 Williams (n 59)

65 M Rabin, ‘Psychology and Economics’ (1998) 36 Journal of Economic Literature 11.

66 See further D. Kahneman, SP Slovic and A Tversky (eds), *Judgment Under Uncertainty: Heuristics and Biases* (CUP 1982).

67 O Seizov, AJ Wulf and J Luzak, ‘The Transparent Trap: A Multidisciplinary Perspective on the Design of Transparent Online Disclosures in the EU’ (2019) 42 Journal of Consumer Policy 149–73.

68 O Ben-Shahar and CE Schneider, *More Than You Wanted to Know: The Failure of Mandated Disclosure* (Princeton UP 2014), emphasis in original.

69 NV Ezechukwu, ‘Consumer-Generated Reviews: Time for Closer Scrutiny?’ (2020) 40 Legal Studies 630–50.

70 TL Beauchamp, ‘Informed Consent: Its History, Meaning, and Present Challenges’ (2011) 20 Cambridge Quarterly of Healthcare Ethics 515–23; Epstein (n 22).

71 J Montgomery, ‘Patient no longer? What next in Healthcare Law?’ (2017) 70 Current Legal Problems 73–109.

72 Arvind and McMahon (n 8). See also AL Caplan, ‘Why Autonomy Needs Help’ (2014) 40 Journal of Medical Ethics 301–2; A Bhangu and others, ‘Is Informed Consent Effective in Trauma Patients?’ (2008) 34 Journal of Medical Ethics 780–82.

73 G Gigerenzer and others, ‘Helping Doctors and Patients Make Sense of Health Statistics’ (2007) 8 Psychological Science in the Public Interest 53–96.

74 KJ Armstrong and others, ‘Effect of Framing as Gain Versus Loss on Understanding and Hypothetical Treatment Choices: Survival and Mortality Curves’ (2002) 22 Medical Decision Making 76–83.

75 PK Han, ‘Conceptual, Methodological, and Ethical Problems in Communicating Uncertainty in Clinical Evidence’ (2013) 70 (1 Suppl) Medical Care Research and Review 14S–36S.

76 ibid.

77 ibid, emphasis in original.

78 AL Simpkin and KA Armstrong, ‘Communicating Uncertainty: A Narrative Review and Framework for Future Research’ (2019) 34 Journal of General Internal Medicine 2586–91.

79 JS Barclay, LJ Blackhall and JA Tulsky, ‘Communication Strategies and Cultural Issues in the Delivery of Bad News’ (2007) 10 Journal of Palliative Medicine 958–77.

80 AJ Lloyd, ‘The Extent of Patients’ Understanding of the Risk of Treatments’ (2001) 10 BMJ Quality & Safety i14–i18.

81 Gigerenzer and others (n 73); C Douglas and E Proudfoot, ‘Nudging and the Complicated Real Life of “Informed Consent”’ (2013) 13 The American Journal of Bioethics 16–17.

82 See eg *Ollosson v Lee* [2019] EWHC 784 (QB).

83 JN Batten and others, ‘What Does the Word “Treatable” Mean? Implications for Communication and Decision-Making in Critical Illness’ (2019) 47 Critical Care Medicine 369.

84 Hallowell (n 16).

85 N Levy, ‘Forced to be Free? Increasing Patient Autonomy by Constraining It’ (2014) 40 Journal of Medical Ethics 293–300. See also Epstein (n 22).

86 JS King and BW Moulton, ‘Rethinking Informed Consent: The Case for Shared Medical Decision-Making’ (2006) 32 American Journal of Law & Medicine 429–501.

87 PH Schuck, ‘Rethinking Informed Consent’ (1994) Yale Law Journal 899–959; JL Madeira, ‘The ART of Informed Consent: Assessing Patient Perceptions, Behaviors, and Lived Experience of IVF and Embryo Disposition Informed Consent Processes’ (2015) 49 Family Law Quarterly 7–28.

88 MA Jacob, ‘Form‐Made Persons: Consent Forms as Consent’s Blind Spot’ (2007) 30 PoLAR: Political and Legal Anthropology Review 249–68; S Cohen, ‘The Gettier Problem in Informed Consent’ (2011) 37 Journal of Medical Ethics 642–45.

89 C Schneider, ‘Some Realism about Informed Consent’ (2005) 145 The Journal of Laboratory and Clinical Medicine 289–91.

90 A Fagerlin, BJ Zikmund-Fisher and PA Ubel, ‘Cure Me Even If It Kills Me: Preferences for Invasive Cancer Treatment’ (2005) 25 Medical Decision Making 616–19.

91 R Sauder and LS Parker, ‘Autonomy’s Limits: Living Donation and Health-Related Harm’ (2001) 10 Cambridge Quarterly of Healthcare Ethics 399–401.

92 Jacob (n 88). See also R Heywood, A Macaskill and K Williams, ‘Patient Perceptions of the Consent Process: Qualitative Inquiry and Legal Reflection’ (2008) 24 Journal of Professional Negligence 104–21; Habiba and others (n 37); JL Madeira and B Andraka-Christou, ‘Paper Trails, Trailing Behind: Improving Informed Consent to IVF through Multimedia Applications’ (2016) 3 Journal of Law and the Biosciences 2–38.

93 M Dixon-Woods and others, ‘Why do Women Consent to Surgery, Even When They Do Not Want To? An Interactionist and Bourdieusian Analysis’ (2006) 62 Social Science & Medicine 2742–53.

94 Habiba and others (n 37).

95 N Sivanadarajah and others, ‘Informed Consent and the Readability of the Written Consent Form’ (2017) 99 The Annals of The Royal College of Surgeons of England 645–49.

96 M O’ Donnell, B Monz and S Hunskaar, ‘General Preferences for Involvement in Treatment Decision Making among European Women with Urinary Incontinence’ (2007) 64 Social Science & Medicine 1914–24.

97 DB Matthew, ‘Race, Religion and Informed Consent – Lessons from Social Science’ (2008) 36 The Journal of Law, Medicine & Ethics 150–73.

98 ibid; Ipsos Mori, *Attitudes Towards Consent and Decision-Making: Prepared for the General Medical Council* (Ipsos Mori 2018).

99 LJ Blackhall and others, ‘Bioethics in a Different Tongue: The Case of Truth-Telling’ (2001) 78 Journal of Urban Health 59–71.

100 Arvind and McMahon (n 8).

101 ibid. S Joffe and others, ‘What Do Patients Value in Their Hospital Care? An Empirical Perspective on Autonomy Centred Bioethics’ (2003) 29 Journal of Medical Ethics 103–8;); R.D. Truog, ‘Expanding the Horizon of Our Obligations in the Clinician-Patient Relationship’ (2017) 47 Hastings Center Report 40-41; Hallowell (n 16).

102 Joffe and others, ibid. NW Dickert and NE Kass, ‘Understanding Respect: Learning from Patients’ (2009) 35 Journal of Medical Ethics 419–23.

103 LR Kass, ‘Practicing Ethics: Where’s the Action?’ (1990) 20 Hastings Center Report 5–12.

104 NC Manson, ‘Why do Patients Want Information If not to Take Part in Decision Making?’ (2010) 36 Journal of Medical Ethics 834–37.

105 O O’Neill, ‘Some Limits of Informed Consent’ (2003) 29 Journal of Medical Ethics 4–7.

106 Ben-Shahar and Schneider (n 68).

107 Manson (n 104).

108 JL Scully, ‘Disability and Vulnerability: On Bodies, Dependence, and Power’ in C Mackenzie, W Rogers and S Dodds (eds), *Vulnerability: New Essays in Ethics and Feminist Philosophy* (OUP 2014) 204–21.

109 JL Madeira, ‘The ART of Informed Consent: Assessing Patient Perceptions, Behaviors, and Lived Experience of IVF and Embryo Disposition Informed Consent Processes’ (2015) 49 Family Law Quarterly 7–28.

110 Ben-Shahar and Schneider (n 68).

111 Levy (n 85).

112 ibid.

113 Bar-Gill (n 14).

